# Primary Sarcoma of the Specialised Prostatic Stroma: A Case Report and Review of the Literature

**DOI:** 10.1155/2011/252805

**Published:** 2011-12-13

**Authors:** Rosanna Zamparese, Francesco Corini, Antonio Braccischi, Antonella D'Angelo, Lucilla Diamanti, Marina Del Vecchio, Willy Giannubilo, Vladimiro Mambelli

**Affiliations:** ^1^Departments of Pathology, General Hospital C.G. Mazzoni, 63100 Ascoli Piceno, Italy; ^2^Department of Urology, Central Hospital, 60035 Jesi, Italy

## Abstract

Primary sarcoma tumours of the prostate are rare and are classified, according to their histology, as stromal tumours of uncertain malignant potential (STUMP) and stromal prostatic sarcoma (PS; low and high grade). We describe a case of a 71-year-old man that developed progressive urinary obstruction symptoms and was subjected to a transurethral prostatic resection (TURP). Histologically, there is a diffuse proliferation of epithelioid and spindle cells that showed rare atypical mitotic figures. Immunohistochemically, the neoplastic cells express diffusely CD34 and focally progesterone whereas no immunoreactivity was seen for cytocheratin, desmin, S-100, Bcl-2, chromogranin, CD117, and actin smooth muscle. A final diagnosis of low-grade prostatic stromal sarcoma (LG-PS) was made. This is a really rare neoplasm; in the literature, in fact, to our knowledge, only 6 cases are described and all of these were alive and free of disease at followup. Our patient too is free of disease at 15 months from the diagnosis.

## 1. Introduction

Malignant tumours of the prostate other than carcinomas are rare [1], among these primary sarcomas are the most frequently observed. 

Primary sarcoma tumors of the prostate arise from specialized hormone-dependent mesenchymal cells [2] and are classified, according to their histology, as stromal tumours of uncertain malignant potential (STUMP) and stromal prostatic sarcoma (PS; namely low grade and high grade) [3–5].

We describe a rare case of low-grade stromal sarcoma (LG-PS) of the specialised prostatic stroma, and we also review the literature concerning these tumours.

## 2. Case Report

A 71-year-old man developed progressive urinary obstruction symptoms and presented at our Hospital. Clinical examination suggested benign prostatic hyperplasia and a transurethral prostatic resection (TURP) was performed.

Macroscopically, the excised tissue consist of many fragments of prostatic tissue weighing 30 grams.

**Figure 1:**
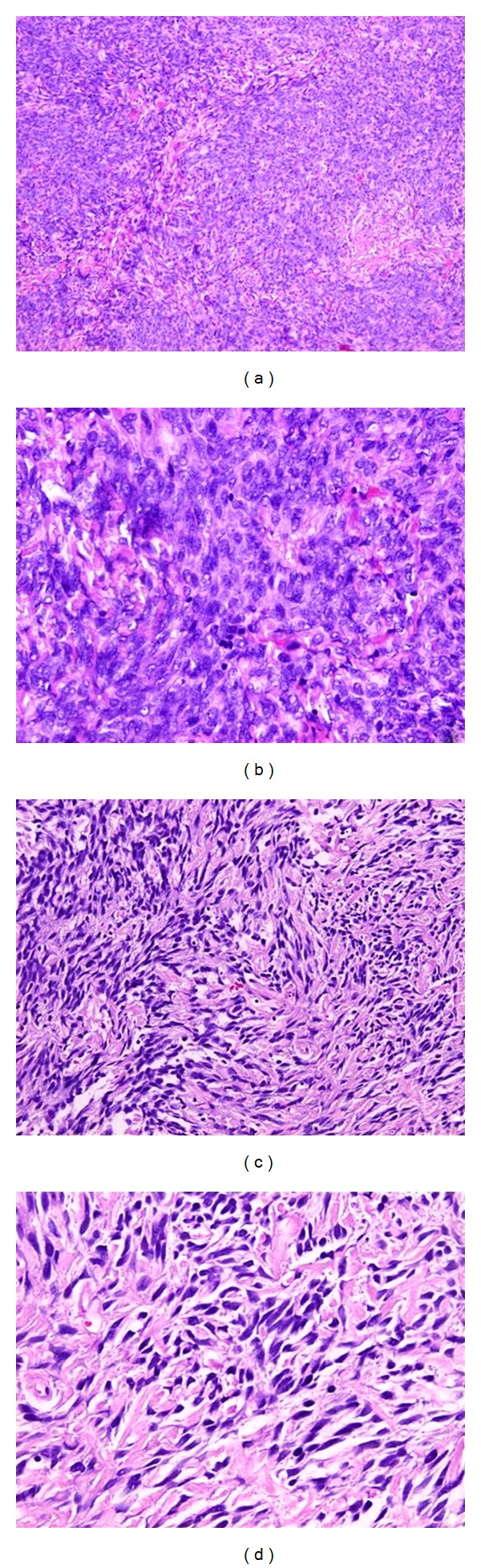
Histological features of low-grade prostatic sarcoma. Diffuse proliferation of *epithelioid* cells ((a) (10 XHPF) and (b) (40 XHPF)) and *spindle* cells, with a storiform and infiltrative growth pattern ((c) (20 XHPF) and (d) (40 XHPF)).

Microscopically, the histological specimens taken after TURP showed a diffuse proliferation of epithelioid and spindle cells, with a storiform and infiltrative growth pattern. There are not prostatic glands in the proliferation, no foci of necrosis, no vascular invasion. The neoplastic cells showed rare atypical mitotic figures in addition to moderate hypercellularity and moderate nuclear atypia with pleomorphism (Figure 1).

**Figure 2:**
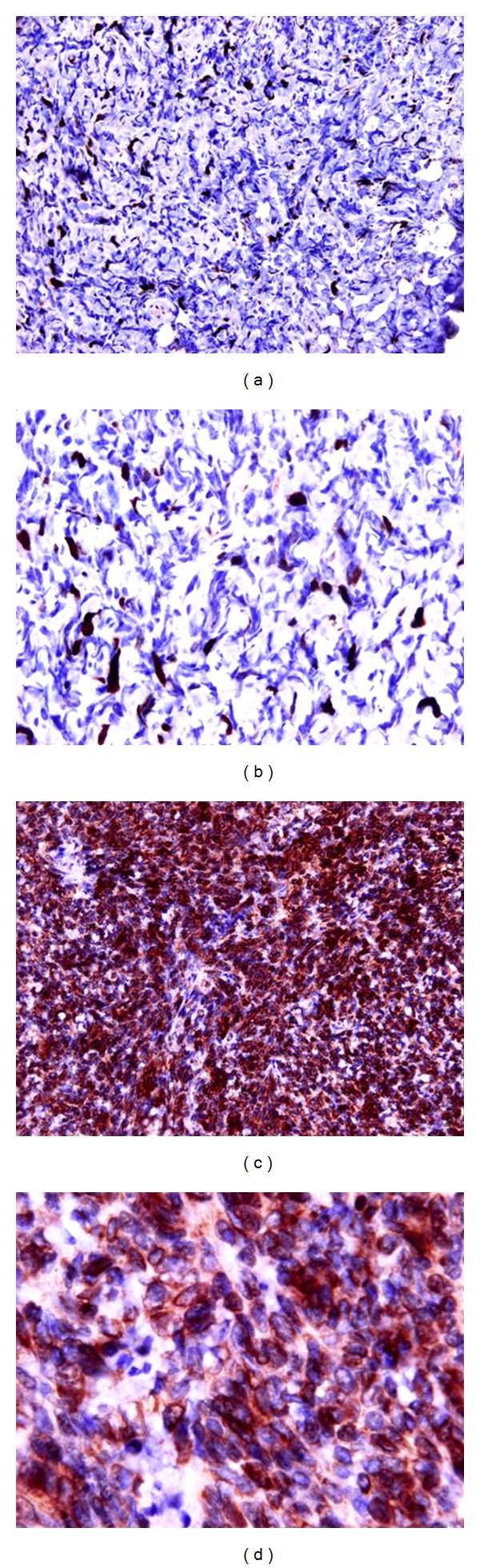
Immunohistochemical expression of CD34 and Ki67. Ki-67 showed a very low expression ((a) (20 XHPF) and (b) (40 XHPF)). The neoplastic cells express diffusely CD34 immunoreactivity ((c) (20 XHPF) and (d) (40 XHPF)).

Immunohistochemically the neoplastic cells characteristically express diffusely CD34 and focally progesterone whereas no immunoreactivity was seen for cytocheratin (clone AE1–AE3 and Cam 5.2), desmin, S-100, Bcl-2, chromogranin, CD117, and actin smooth muscle (Figure 2).

We evaluated additional immunohistochemical markers for their potential diagnostic and therapeutic utility. Although p53 and Ki-67 showed a very low expression.

On the basis of the morphological and immunohistochemical features, a final diagnosis of LG-PS was made.

## 3. Discussion

Spindle cell lesions of the prostate encompass a broad spectrum of benign and malignant processes. Some are characteristic of this organ and may be benign or malignant, including leiomyoma and leiomyosarcoma, rhabdomyosarcoma, inflammatory myofibroblastic tumour, solitary fibrous tumours, phyllodes tumour, and synovial sarcoma [3–7].

Prostatic stromal tumors, arising from the specialized prostatic stroma, are rare and distinct tumours with diverse histologic pattern. In the past, these tumors have been reported under a variety of terms including atypical stromal hyperplasia, phyllodes type of atypical stromal hyperplasia, and cystic epithelial-stromal tumors [5]. Now these lesions have been classified as lesions of uncertain malignant potential (STUMP) and stromal sarcoma (low grade and high grade) [6].

Because of their rarity and the nonspecific nature of clinical symptoms, their diagnosis is rarely suspected clinically.

Histologically the diagnosis of HG-PS is based on observation of a neoplasm with a proliferation of epithelioid and spindle cells that showed different growth patterns (solid, storiform, epithelioid, short fascicles, spindled, rhabdoid, and malignant phyllode-like). The neoplastic cells showed marked nuclear atypia, mitosis, and necrosis [5]. LG-PS, instead, showed no nuclear atypia and low mitotic rates, can locally invade, despite having relatively bland cytology at times. HG-PS has the potential to be metastasized.

In contrast to stromal sarcoma, the neoplastic nature of STUMPs is controversial. STUMPs may be considered neoplastic based on the observation that they may diffusely infiltrate the prostate gland and extend into adjacent tissues [8, 9].

In most cases STUMP is not of an aggressive nature, occasional cases have been documented with an extension into adjacent tissues or recurrence after resection [10].

The predominant pattern of STUMP consists of either normocellular or mildly hypercellular stroma with cytologically degenerative atypical cells associated with benign glands. The currently used classification of STUMP was first proposed in 1998 by Gaudin et al. [4], in which 4 patterns of stroma were described: degenerative atypia, cellular stromal pattern, phyllodes pattern, and stromal predominant/myxoid pattern. As was the case in the initial and subsequent series on STUMP, the degenerative atypia subtype was the most common pattern seen [5].

The epithelial component proliferations occurring within the STUMP, only recently described [11], is histologically normal in 17% of cases, and there is a tendency toward urothelial and squamous metaplasia in STUMPs with a phyllodes pattern, and a prominent basal cell layer in STUMPs with degenerative and cellular stroma [11].

In the past, the histologically pattern of STUMP was often reported as benign prostatic hyperplasia (BPH) with atypia and in rare cases may be difficult to distinguish form stromal sarcoma [5]. Although STUMP can be histologically misdiagnosed as benign prostatic hyperplasia, it is important to recognized that these are neoplasms with unique local morbidity and malignant potential. In contrast to BPH, STUMP can recur frequently and occur at younger men.

Sometimes high- and low-grade prostatic stromal sarcoma was associated with STUMP. Herawi and Epstein, in fact, found that seven (14%) of the 50 stromal tumours of the prostate that they analyzed were STUMPs associated with sarcoma (4 HG-PS, 3 LG-PS) [5]. 

There are several difficulties with the histologic diagnosis of STUMP, LG-PS, and HG-PS; it may be difficult to differentiated LG-PS from STUMP, sometimes the tumour seemed histologically benign and only the presence of local infiltration or the presence of atypical mitotic figure was diagnostic of LG sarcoma.

Prognosis and treatment of STUMP and sarcoma are also controversial. Factors to consider in deciding if to proceed with definitive resection for STUMP diagnoses on biopsy include patient's age, presence and size of the lesion, and extent of the lesion, on tissue sampling.

Immunohistochemically, STUMP and stromal sarcomas characteristically express progesterone receptors (PR), and this finding is consistent with their arising from hormonally responsive mesenchymal cells of the prostate [12, 13]. 

 Most of these tumors are positive for CD34 [4]. The immunohistochemically strong positivity of CD34 and the negativity of desmin is helpful in the differential diagnosis of STUMP and stromal sarcoma versus smooth muscle tumours [14]. Rarely, gastrointestinal stromal tumors (GIST) may enter the differential diagnosis, but these tumours are CD117 positive while stromal sarcomas are negative [5]. 

The primary prostatic sarcoma are really rare neoplasms. In the literature, to our knowledge, only 6 cases are described of LG-PS (age: 42 to 76, mean 59; see Table 1), except phyllodes tumour [5–15], and between these cases only three are pure sarcoma, the others are associated with a STUMP. All of the patients with LG-PS were alive and free of disease at followup [5–15]. 

In the current literature only 16 cases are also reported of HG-PS [5–15].

The HG-PS are more aggressive tumour, in fact only 6 patients of 16 cases, reported in the literature, were free of disease, 3 were alive with pulmonary metastases, 2 were alive with multiple metastases, and 3 died of disease and 2 were lost to followup [5–15].

Our case of LG-PS is the seventh that was described in the literature, and our patient was free of disease at 15 months of followup.

## Figures and Tables

**Table 1:** Review of The literature of low-grade prostatic sarcoma.

Authors	Age (years)	Size (cm) weigh (g)	Association with STUMP	Followup
Herawi and Epstein [5]	75	988 g	Yes	No evidence of disease at 13 months from diagnosis
	63	2.5 cm	Yes	No evidence of disease at 2 years from diagnosis
	76	10 g	Yes	No evidence of disease at 25 months from diagnosis
	42	2.5 cm	No, pure sarcoma	No evidence of disease at 30 months from diagnosis
	43	4 cm	No, pure sarcoma	No evidence of disease at 36 months from diagnosis
	43	2.7 cm	No, pure sarcoma	No evidence of disease at 3 months from diagnosis

Current paper	71	30 g	No, pure sarcoma	No evidence of disease at 15 months from diagnosis
